# Supplementary material for: GeNET: a web application to explore and share Gene Co-expression Network Analysis data
Source: PeerJ. 2017 Aug 14;5:e3678. doi: 10.7717/peerj.3678 (PMC5560228; doi:10.7717/peerj.3678)
Supplement: Supplemental Information 1 — Tutorial document explaining how to find new genes of interest using GeNET, and how to upload GCNA data into GeNET. [file peerj-05-3678-s001.pdf]

GeNET's how-tos

How to find new genes of interest?

1. **If you have a pathway or function of interest** and want to find new genes that may be related to this pathway or function, then go to GeNET's main page, select your organism of interest, click `Search Pathway`, enter a keyword for your pathway/function of interest (alternatively you can enter * to see all over-represented pathways/functions), and click `Go`. Click on the module enriched with genes annotated to your pathway/function of interest. On GeNET's module-centric view, browse the genes in the module, and the module graph view to identify potential genes of interest. Once a potential gene of interest has been identified, visit that gene's page on GeNET for more information about that gene.

2. **If you have an experimental condition of interest** and want to find new genes that might be up or down in that condition, then go to GeNET's main page, select your organism of interest and click on `Browse Module`. In the Module Browser, click on the conditions of interest. Co-expression modules with an average gene expression significantly up or down in those conditions are highlighted. You can then visit the module-centric view for those modules to identify potential genes of interest. Once a potential gene of interest has been identified, visit that gene's page on GeNET for more information about that gene.

3. **If you have a gene of interest** and want to find new genes that might be functionally related to this gene, then go to GeNET's main page, select your organism of interest and click on `Search Gene`, select the type of identifier (either Symbol, Entrez ID or Systematic ID) to use and enter the gene identifier on the text box (GeNET will display options for autocompletion). Click `Go` to visit the corresponding gene-centric view. On the gene-centric view, go to the graph representation and use the `Correlation filter` (located on the right side of the page) to identify genes whose expression profile is highly correlated to your gene's expression profile. Highly correlated genes will have an edge to the gene of interest in the graph. Once a potential gene of interest has been identified, visit that gene's page on GeNET for more information about that gene.

How to find a gene in GeNET if the gene identifier I have is not supported by GeNET?

For most organisms there are multiple gene identifiers (e.g., RefSeq IDs, Entrez IDs, Ensembl IDs, organism-specific IDs, etc). GeNET supports three identifiers per gene. One has to be Entrez IDs and the other two are the choice of the data contributor. If you have an identifier for your gene of interest which is not supported by GeNET, you can use ID mapping tools to find out the corresponding gene identifier supported by GeNET.

1. For rice, there is an ID converter available at http://rapdb.dna.affrc.go.jp/tools/converter
2. For other organisms, the following web tools allow to map between a wide variety of gene identifiers: http://www.uniprot.org/uploadlists/ and https://david.ncifcrf.gov/conversion.jsp

How to prepare data to upload into GeNET?

1. Find out the NCBI taxonomy ID of your organism, and the PubMed ID of the publication describing the GCNA analysis.
2. Create a GENE_INFO table with the Entrez ID, Symbol, other ID, description, biological type and co-expression module for all genes in the corresponding organism. The other ID could be any gene ID of your choice (e.g., ENSEMBL ID, UniProt ID, RefSeq ID, etc). Biological type indicates the type of each gene, for example, protein-coding, tRNA, miRNA, etc. The Symbol should uniquely represent one gene (i.e., a Symbol should only appear in a single row in the table) and should not contain spaces or brackets. The table should contain a header and be tab-delimited.
3. Create a CONDITION_INFO table with the ID, number of replicates, strain, growth, medium and description for all experimental conditions included in the gene expression data. The ID must uniquely represent one experimental condition and should not contain spaces or brackets. The strain, growth, medium and description should describe the experimental condition. Each of these columns may contain up to 250 characters. The table should contain a header and be tab-delimited.
4. Create a GENE_EXPRESSION table with the normalized gene expression measurements for each gene / condition pair. This table should contain the gene expression data used to perform the gene co-expression network analysis (GCNA). Columns are the IDs of the experimental conditions and rows are the Symbols of the genes. Make sure all the condition IDs and gene Symbols appear in the CONDITION_INFO and the GENE_INFO table respectively. NAs are allowed to represent missing values. Table should be tab-delimited.
5. OPTIONAL. Create a GENE_ANNOTATION table with the gene Symbol and the corresponding annotation (e.g., GO terms, pathway IDs, co-regulated gene sets, PFAM protein domains, etc.). Make sure all the gene Symbols in this table appear in the GENE_INFO table. The table should contain a header and be tab-delimited.
6. OPTIONAL. Create a FASTA file with the protein sequences of your organism. The headers of the FASTA file should be the gene Symbols.

How to upload data into GeNET?

1. Go to GeNET's Data Contributor Login at http://bengi.cs.mun.ca/genet/login
2. If you don't have a GeNET account, create an account. Login into GeNET.
3. Fill the form shown below by:
    1. Entering the NCBI taxonomy ID for your organism
    2. Entering the PubMed ID for the article describing the GCNA results
    3. Selecting the text file containing the GENE_EXPRESSION table
    4. Indicating the parameters used for the GCNA
    5. Selecting the text file containing the GENE_INFO table
    6. Selecting the text file containing the CONDITION_INFO table
    7. Entering at least one of the following:
        1. The KEGG ID for your organism
        2. A text file with a GENE_ANNOTATION table
        3. A text file with a GENE_ANNOTATION table associating the genes with PFAM protein domains or a FASTA file with the protein sequences of your organism.

    GeNET allows up to five sources of functional annotation: KEGG pathways, PFAM protein domains and three other GENE_ANNOTATION tables containing functional annotation of your

choice.

8. Agreeing to GeNET's privacy and data publication policy available at http://bengi.cs.mun.ca/genet/contact#privacy

4. Click on `Submit`.
5. You will receive an email confirming your submission, and then an email notifying the results of the upload.

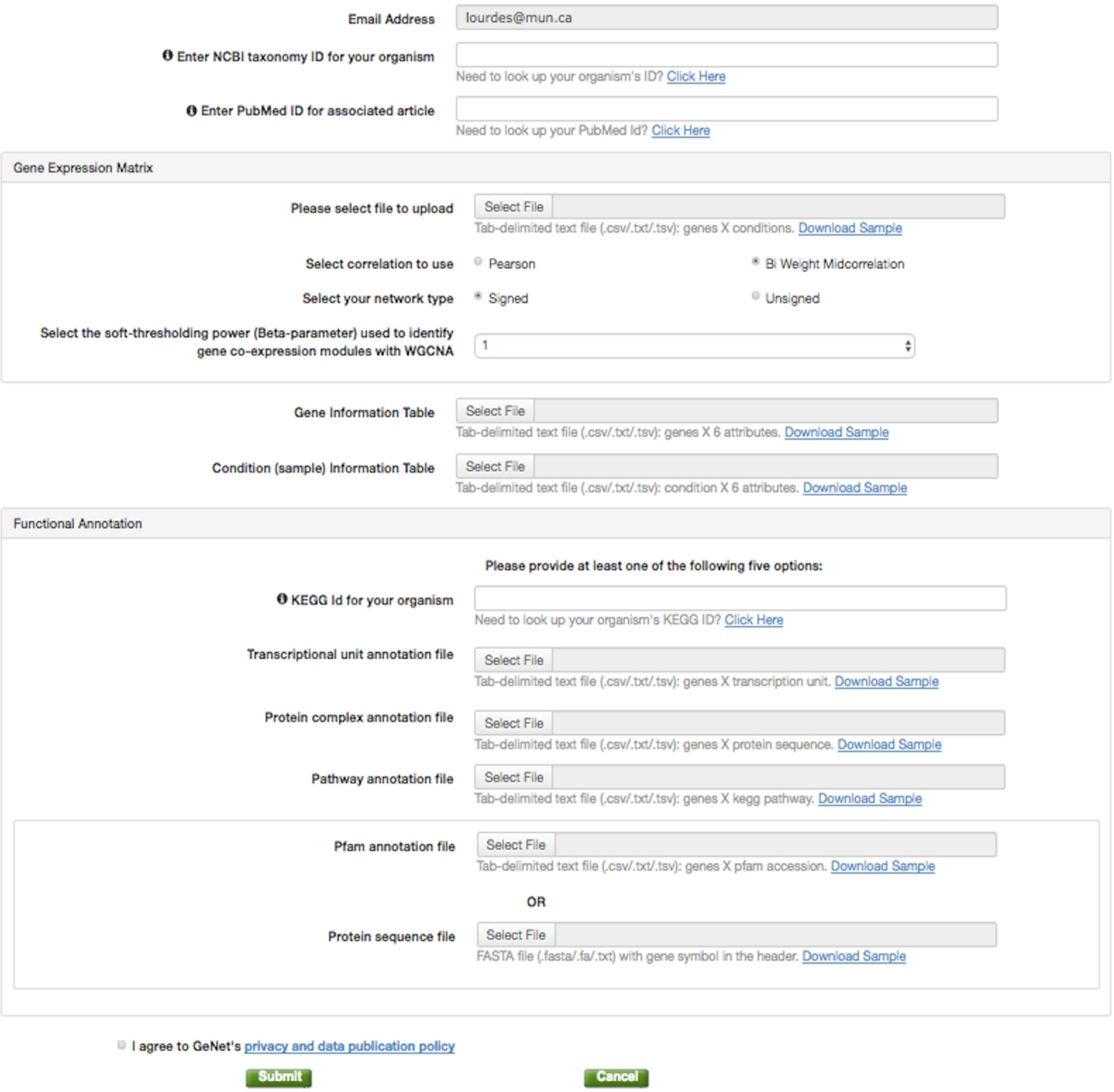
Email Address: lourdes@mun.ca

Enter NCBI taxonomy ID for your organism
Need to look up your organism's ID? Click Here

Enter PubMed ID for associated article
Need to look up your PubMed Id? Click Here

Gene Expression Matrix

Please select file to upload: Select File
Tab-delimited text file (.csv/.txt/.tsv): genes X conditions. Download Sample

Select correlation to use: Pearson / Bi Weight Midcorrelation

Select your network type: Signed / Unsigned

Select the soft-thresholding power (Beta-parameter) used to identify gene co-expression modules with WGCNA: 1

Gene Information Table: Select File
Tab-delimited text file (.csv/.txt/.tsv): genes X 6 attributes. Download Sample

Condition (sample) Information Table: Select File
Tab-delimited text file (.csv/.txt/.tsv): condition X 6 attributes. Download Sample

Functional Annotation

Please provide at least one of the following five options:

KEGG Id for your organism
Need to look up your organism's KEGG ID? Click Here

Transcriptional unit annotation file: Select File
Tab-delimited text file (.csv/.txt/.tsv): genes X transcription unit. Download Sample

Protein complex annotation file: Select File
Tab-delimited text file (.csv/.txt/.tsv): genes X protein sequence. Download Sample

Pathway annotation file: Select File
Tab-delimited text file (.csv/.txt/.tsv): genes X kegg pathway. Download Sample

Pfam annotation file: Select File
Tab-delimited text file (.csv/.txt/.tsv): genes X pfam accession. Download Sample

OR

Protein sequence file: Select File
FASTA file (.fasta/.fa/.txt) with gene symbol in the header. Download Sample

I agree to GeNet's privacy and data publication policy

Submit Cancel
